# Toward Smart Diagnostics in a Pandemic Scenario: COVID-19

**DOI:** 10.3389/fbioe.2021.637203

**Published:** 2021-06-17

**Authors:** Mohammad Hosseinifard, Tina Naghdi, Eden Morales-Narváez, Hamed Golmohammadi

**Affiliations:** ^1^Nanosensor Bioplatforms Laboratory, Chemistry and Chemical Engineering Research Center of Iran, Tehran, Iran; ^2^Biophotonic Nanosensors Laboratory, Centro de Investigaciones en Óptica, Guanajuato, Mexico

**Keywords:** biosensors, point-of-care testing, internet of things, artificial intelligence, smartphone

## Abstract

The incredible spread rate of coronavirus disease 2019 (COVID-19) outbreak has shocked the world. More than ever before, this dramatic scenario proved the significance of diagnostics as a cornerstone to make life-saving decisions. In this context, novel diagnostics that generates smart data leading to superior strategies for treatment, control, surveillance, prediction, prevention, and management of pandemic diseases is vital. Herein, we discuss the characteristics that should be met by COVID-19 diagnostics to become smart diagnostics enabled by industry 4.0 especially Internet of Things (IoT). The challenges ahead and our recommendations for moving faster from pure diagnostics toward smart diagnostics of COVID-19 and other possible epidemic/pandemic diseases are also outlined. An IoT-Fog-Cloud model based on smartphones as IoT gateways for smart diagnostics with unified strategies for data collection/transmission/interpretation is also proposed to integrate new digital technologies into a single platform for smarter decisions. Last but not least, we believe that “smart diagnostics” is a perspective that should be realized sooner before we encounter a pandemic far worse than the present one.

## Introduction

Since the first reports of the coronavirus disease 2019 (COVID-19) outbreak on 31st December 2019 in Wuhan, China, when many of us believed that the overall wellbeing had been improved by current scientific, technological and industrial achievements, the rapid and unbelievable spread rate of severe acute respiratory syndrome coronavirus 2 (SARS-CoV-2) pandemic has shocked the whole world. COVID-19 severely affected the health, economy, and social activities of more than seven billion people worldwide (Callaway, 2020). Since the last days of 2021 up to March 2021, over 116,166,652 people have been infected with this disease and unfortunately, over 2,582,528 have lost their lives all over the world [World Health Organization (WHO) Situation Report, 7th March 2021] (World Health Organization, 2021).

It was recently estimated that COVID-19 pandemic economic impact could reach between $5.8–$8.8 trillion globally which is equal to 6.4–9.7% of global gross domestic product (GDP) (Park et al., 2020). Apart from other non-measurable effects mentioned above (i.e., social/political), such high economic damage and mortality/morbidity rate must be enough motivation for establishing some new rules/changes, methods, and safety/active measures to prevent them from happening or at least fight them smartly.

Accurate, precise, timely, and widespread diagnosis of potentially pandemic outbreaks can be considered as the first effective measure to make life-saving smart decisions in order to start a timely process of treatment, trace and track infected people to isolate each possible suspect, thereby, controlling and breaking its transmission chain (Cohen and Kupferschmidt, 2020). A well-proven experience has been demonstrated by countries such as Germany, Singapore, and South Korea, which performed a large number of COVID-19 diagnostic tests in a widespread and effective manner in the early days of awareness of this pandemic outbreak (Lee and Lee, 2020; Morales-Narváez and Dincer, 2020; Ting et al., 2020). This measurement resulted in effective regional control and management of the disease and subsequently saved the lives of many citizens of these countries (Cohen and Kupferschmidt, 2020). However, the ubiquitous character of COVID-19 not only requires easy access to high-quality tests all over the world but also some smart strategies to cope with this pandemic situation globally. In this context, we believe that smart diagnostics can play a critical role in the fight against pandemics and also in the prediction and prevention of future epidemics or pandemics.

Smart diagnostics can be defined as a diagnosis based on smart data resulted/analyzed from diagnostic tests/devices’ big data. This concept spans diagnostics interconnected with powerful resources facilitated by Industry 4.0 in the form of new digital technologies such as Internet of things (IoT), smartphone, big data analytics (BDA), machine learning (ML), deep learning (DL), blockchain analysis (BA), artificial intelligence (AI), augmented reality (AR), cybersecurity, system integration, cloud, and fog computing (Ting et al., 2020). These smart tools lead to a myriad of capabilities to address pandemic scenarios, from generation of biomedical data to resourceful strategies for treatment, control, surveillance, prevention, and management of pandemic diseases. For example, point-of-care (POC) devices (Dincer et al., 2019) can be interconnected with real-time online databases, epidemiologic modeling (potential growth and areas of spread), virtual clinics, AI-assisted diagnostics, and prognostics to automatically classify medical conditions, as well as mechanisms related to the distribution of patients’ medications to the local pharmacies or patients’ doorsteps (Ting et al., 2020).

In this perspective, we aim to highlight the necessity of smart diagnostics in a pandemic scenario. We underscore the vital role of smart diagnostics in aid to overcome the present pandemic globally and predict and subsequently prevent the next possible epidemics/pandemics. We also consider an overview of COVID-19 diagnostics to discuss the criteria that should be met by the developed diagnostic tests/devices, as well as new digital technologies related to smart diagnostics. Eventually, future challenges/strategies and our recommendations for future diagnostic tests/devices to fulfill the smart diagnostics of infectious disease outbreaks are offered.

## Toward Smart Diagnostics of COVID-19

COVID-19 displays an incubation period ranging from 2 to 14 days, with symptoms appearing from 8 to 16 days after infection exposure (Weissleder et al., 2020). Individuals infected by COVID-19 show different symptoms, which vary from asymptomatic infection to severe respiratory failure (He et al., 2020). However, common symptoms of SARS-CoV-2 infections include fever, cough, fatigue, slight dyspnoea, sore throat, headache, and conjunctivitis. Given the development of cold/flu-like symptoms, COVID-19 can be confused with other respiratory illnesses, including those provoked by influenza or rhinoviruses (Pascarella et al., 2020; Ting et al., 2020). Hence, highly sensitive and specific COVID-19 testing is the cornerstone to address this ambiguity.

Given the crucial role of testing in the treatment and isolation of COVID-19 infected people, several COVID-19 diagnostic methods have been developed all over the world throughout 2020 (Pokhrel et al., 2020). Furthermore, COVID-19 testing is also crucial to control and prevent possible upcoming SARS-CoV-2 outbreaks. Emerging approaches, operational principles, detailed technical descriptions, sensitivity, and specificity related to COVID-19 diagnostic methods have been eloquently elaborated by different groups (Carter et al., 2020; Cui and Zhou, 2020; Ji et al., 2020; Pascarella et al., 2020; Sheridan, 2020; Udugama et al., 2020; Weiss et al., 2020; Weissleder et al., 2020).

On balance, we can find two main targets related to COVID-19 diagnostics, including (i) SARS-CoV-2 genetic material and (ii) immunoglobulins, produced after COVID-19 infection. Generally, acute infection of the novel coronavirus can be detected through its genetic material in nasopharyngeal swab samples using conventional methods such as reverse transcription-polymerase chain reaction (RT-PCR) or employing novel methods, including reverse transcription- loop mediated isothermal amplification (RT-LAMP) and clustered regularly interspaced short palindromic repeats (CRISPR)-based technology (Morales-Narváez and Dincer, 2020; Weissleder et al., 2020). COVID-19 acute infection can also be diagnosed *via* SHERLOCK (specific high-sensitivity enzymatic reporter unlocking) and thoracic imaging using computed tomography (CT) scans (Joung et al., 2020). Moreover, SARS-CoV-2 seroconversion in humans involves the production of antibodies approximately 7 days after the onset of the disease and this antibody response is stable at least 3 months (Wajnberg et al., 2020). SARS-CoV-2 seroconversion interrogation is not particularly useful to determine COVID-19 infection at the onset of the disease, but it is crucial to determine the transmission and immunity of COVID-19 infection (Clapham et al., 2020). The resultant antibodies can be detected in blood or serum samples using conventional immunoassays such as enzyme-linked immunosorbent assay (ELISA). In this context, the detection of the genetic material of the novel coronavirus and detection of antibodies generated by SARS-CoV-2 infection are complementary approaches. In other words, generally, diagnostics targeting SARS-CoV-2 genetic material are useful to monitor active cases, whereas diagnostics targeting COVID-19-related immunoglobulins are useful to monitor past infections.

Moreover, the whole virus can be captured and detected in saliva samples using biorecognition elements such as antibodies integrated into biosensing systems (Morales-Narváez and Dincer, 2020). Typically, the aforementioned diagnostics analyzing nasopharyngeal swab samples are carried out by centralized services; however, readily available samples, for example, saliva or a few drops of blood/serum facilitate COVID-19 detection at the POC (Cui and Zhou, 2020). Hence, depending on the nature of the sample, COVID-19 diagnostics can also be performed *via* POC devices incorporating nucleic acid or immunoglobulins testing. In fact, equipment enabling on-site isothermal amplification of the novel coronavirus genetic material and lateral flow (LF) strips (pregnancy-like tests) targeting immunoglobulins resulting from COVID-19 infection are prominent examples of POC devices employed in SARS-CoV-2 diagnostics (Point-of-Care, 2020; Sheridan, 2020).

The sensitivity of rapid COVID-19 tests is affected by various factors including target molecules (ex. genetic material, antigens or antibodies), testing strategy, disease severity, test duration, required equipment, operator skill in sampling/specimen collection, running the tests and reading/interpreting the results, and required transport and storage conditions (Land et al., 2019; Mina et al., 2020; Watson et al., 2020; Crozier et al., 2021).

On the one hand, the developed COVID-19 diagnostic tests/devices are available in the economically developed world– either as centralized services or as POC approaches; on the other hand, most of them are rarely available or affordable in resource-limited settings, such as the developing world and especially resource-limited setting due to their high cost, which is mainly related to the high costs of fabrication, materials, (bio)chemicals, human resources, and equipment used for their development, and other possible costs associated with their transportation/storage/refrigeration, and delivery to end-users (Land et al., 2019). In this context, as discussed above, the pandemic nature of COVID-19 has created new expectations/needs of COVID-19 diagnostic tests/devices. Such expectations/needs are aimed to solve the pandemic scenario by making smart decisions and preventing new pandemic waves through their smart prediction. Therefore, the most important question in the field of design and fabrication of COVID-19 diagnostic tests can be the following one: “What criteria should be met for such ideal diagnostic tests/devices for the smart diagnostics of infectious diseases with high epidemic/pandemic potential”? The answer to this question can be found by reviewing the criteria developed by WHO for ideal diagnostic tests/devices and experiences gained over the past two decades in implementing those diagnostic policies.

Given the extreme importance of early diagnosis in proper prevention and treatment of emerging infectious diseases and saving many lives, especially in developing regions, a set of criteria was published in 2003 by WHO for ideal diagnostic devices/assays (Mabey et al., 2004; Land et al., 2019). According to these criteria, the ideal diagnostic tests/devices that can be utilized in health care systems of the developing countries, including POC applications, should be affordable, sensitive, specific, user-friendly, rapid and robust, equipment-free and deliverable to end-users, which were coined by the acronym ASSURED (Kettler et al., 2004; Peeling et al., 2006). However, the WHO’s experience of more than a decade of implementing ASSURED diagnostics demonstrated that despite many benefits of the developed ASSURED-based diagnostic tests/devices compared to the pre-ASSURED diagnostics period, access to diagnostic tests, which can be performed with high efficiency at the POC, has remained as a critical challenge, especially in the developing countries and resource-limited settings. Besides, during the last decades, along with the development of ASSURED-based diagnostic devices, as displayed in Scheme 1, further progress was being made in other areas such as new digital technologies i.e., smartphones, IoT, AI, ML, BDA, DL, BA, Fog/Cloud computing, microfluidic paper-based analytical devices, and so on (Scarpino and Petri, 2019; Wheeler, 2019; Wu et al., 2020). To incorporate new developed/emerging technologies in the design and fabrication of future diagnostic tests/devices for overcoming the shortcomings of ASSURED diagnostics, strengthening the efficiency of health care systems, informing disease control strategies, and improving patient outcomes, the term REASSURED was coined in 2018 by adding two new criteria of real-time connectivity (R), and ease of specimen collection and environmentally friendly (E) to the former WHO’s ASSURED criteria (Land et al., 2019). In this regard, more than ever before, the COVID-19 pandemic scenario demonstrates the importance and necessity of newly added criteria (RE) in the development of the ideal diagnostic tests/devices of the future.

**SCHEME:**
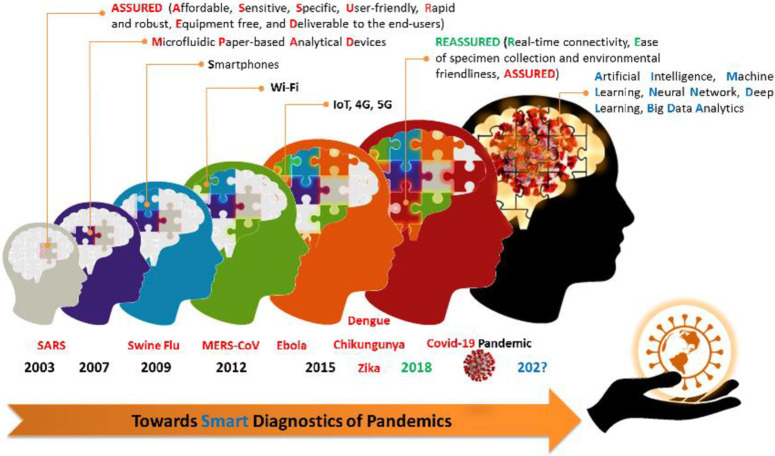
Smart diagnostics, relevant technologies, and infectious diseases timeline.

In a pandemic scenario, real-time connectivity competence is crucial to read and transmit the test results and receive feedback. The use of COVID-19 diagnostic devices with this capability will lead to faster and timelier testing and provide feedback from decision-makers, remote monitoring of the patients’ condition and clinical progress of the treatment process, and subsequently faster and better control of this disease. Besides, this ability will facilitate a significant decrease in turnaround time, energy consumption, carbon-footprint, society/health services cost, and more importantly referring people with suspected COVID-19 (infected or non-infected) to diagnostic centers to perform or obtain test results, which will effectively tackle the transmission chain of this disease, thereby contributing to the control of the spread of the disease. Furthermore, the data obtained from the decentralized real-time connected COVID-19 diagnostic devices will efficiently help to control this disease timely by taking advantage of centralized and real-time decision-making. Importantly, smart prediction can also be supported by these resources to prevent new pandemic waves in the future.

One of the main limitations of most of the developed COVID-19 diagnostic tests/devices is their reliance on the use of invasive specimens and/or difficult and complex specimen collection (such as nasopharyngeal swabs or several milliliters of blood/serum), as well as processing methods that limit their applications at the POC and remote sites away from centralized laboratory facilities. Owing to such difficulties, most of the existing COVID-19 diagnostic tests can only be performed in centralized/sophisticated laboratories with trained operators to run the diagnostic tests and interpret their results, and for this reason, many of them, such as PCR and ELISA, are still far from being used as self/home diagnostic tests.

Apart from the obvious and great importance of ASSURED criteria in the design of ideal COVID-19 diagnostic tests/devices (see Table 1 and Supplementary Material) (Land et al., 2019), another crucial criterion that should be taken into account in a pandemic scenario, involving the need of performing its diagnostic tests in vast geography and great numbers, is to pay attention to the environmental requirements in their fabrication. This criterion is important in such accumulation of large volumes of the used COVID-19 diagnostic tests, which are not environmentally friendly (i.e., the environmental requirements have not been met in their design and fabrication), as they can lead to health and environmental hazards especially in resource-limited settings. We believe that cellulose-based materials and bioplastics are an excellent choice to fabricate REASSURED devices (Golmohammadi et al., 2017; Bhagwat et al., 2020).

**TABLE 1:**
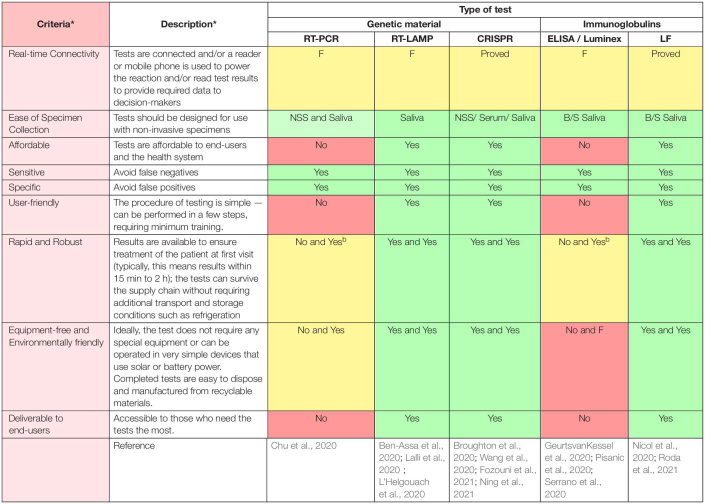
The evaluation of the main COVID-19 diagnostic tests/devices based on REASSURED criteria.

*^a^Adapted from Land et al. (2019). ^b^Generally requires specialized transport and storage conditions. NSS, nasopharyngeal swab sample; B/S, a few drops of blood or serum; F, feasible.

**FIGURE 1:**
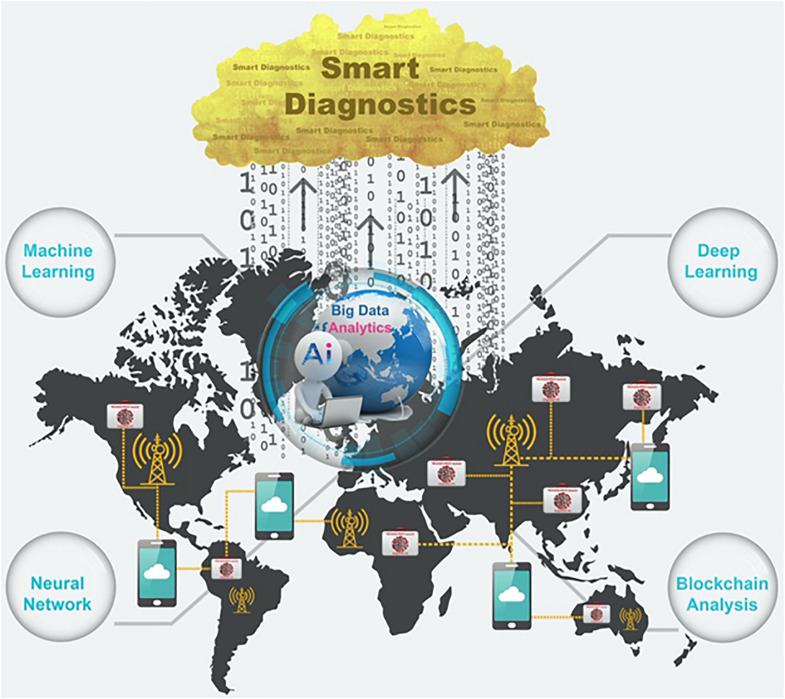
Schematic representation: how to realize smart diagnostics in a pandemic scenario.

As shown in Figure 1, the special advantage of REASSURED diagnostics is that the smart analysis of the data collected from millions of the decentralized REASSURED-based diagnostic devices all over the world through digital technologies such as BDA, ML, DL, neural network (NN), BA, and AI, can eventually lead to “Smart Diagnostics.” As discussed above, in a pandemic scenario, smart diagnostics can be defined as a diagnosis based on smart data resulted/analyzed from REASSURED-based diagnostic tests/devices’ big data.

COVID-19 pandemic demonstrated more than ever the importance of smart diagnostics and consequently the necessity of moving faster the diagnostic tests/devices toward becoming smart. Table 1 displays the main diagnostic technologies targeting COVID-19 and their opportunities in terms of REASSURED criteria. Requirements/criteria yet to be met by each of them toward smart diagnostics of COVID-19 are also underscored in Table 1. To the best of our knowledge, RT-LAMP, CRISPR, and LF technology hold the best opportunities as REASSURED approaches (see Supplementary Material), though real-time connectivity should be implemented and widely demonstrated. For example, real-time connectivity of diagnostic technologies can be supported by quick response (QR) codes (Scherr et al., 2017) with international standards using fog servers with lower latency compared to cloud ones.

Interestingly, smartphones as one of the easy-to-use and ubiquitous—yet efficient and promising—tools can play a significant role in moving from pure sensing to smart sensing and subsequently realizing smart diagnostics. The reason for this can be attributed to the fascinating and unrivaled features of smartphones as miniature computers with fast-operating-systems. 94% of the world’s population (with more than 70% from the developing countries) is presently using smartphones which equals 6.8 billion subscribers connected to the Internet while the number of subscribers is rising (Vashist and Luong, 2019). It means that they are widely-accessible even in resource-limited settings without suitable healthcare services (Xu et al., 2015). While the majority of the world population carries an efficient universal tool in their pockets as “Lab-in-the-Pocket” (Le Page, 2016; Radin et al., 2016), there are unprecedented opportunities for value-added products and services, particularly for connected/real-time diagnostics (Wood et al., 2019) in pandemics and transition from pure diagnostics to smart diagnostics. Besides, the recent advances in the related technologies such as internet connectivity (4/5G), data management (edge/fog/cloud computing), high-resolution cameras, wireless, near-field communication (NFC), Bluetooth, and powerful processors make smartphones as ideal interoperable tools for (bio)sensing/diagnostics purposes allowing communication between decentralized tests/devices and centralized laboratories/data-centers for smarter services. In this context, smartphones can act as IoT gateways *via* interoperability (Aloi et al., 2017) to reach the Internet of Medical Things (IoMT) (Islam et al., 2015) and Internet of Analytical Things (IoAT) (Mayer and Baeumner, 2019) and become mediators for smart diagnostics.

Last but not least, if we put aside the obvious mediating/interoperable role of smartphones for telemedicine and remote patients monitoring *via* numerous IoT devices such as smart thermometers/patches/biosensors (Chamola et al., 2020; Morales-Narváez and Dincer, 2020) for COVID-19, they can share epidemiological/immunological/clinical/genetic data (Xu et al., 2015), considering data security with no privacy violation (Ienca and Vayena, 2020; Morley et al., 2020), and even possible symptoms of COVID-19, for instance, dry cough, tiredness, chest pain, diarrhea, rash, loss of taste or smell and so on, which there is currently no way/tool to assay them other than the user statements. Such feature will empower the users to have an active role in the war against public enemy no. 1 *via* activating them to transmit more sophisticated diagnostic data in form of symptoms to COVID-19 data-centers for further support, smart diagnostics, and precision medicine, which is very promising for breaking the transmission chain and will considerably reduce cost, time, energy, and carbon-footprint during pandemics. As a result, the use of smartphone-connected REASSURED diagnostic tests/devices, due to their ability to send data related to health/symptoms as complementary information along with diagnostic data, can be considered as a strategic approach toward smart diagnostics of COVID-19.

## Final Remarks and Outlook

A part from considering the REASUURED criteria in the fabrication of the COVID-19 diagnostic tests/devices, another important step for realizing smart diagnostics is to design a roadmap and an applicable model that can efficiently integrate all new digital technologies into a single platform with a unified strategy in order to use the data obtained from diagnostic tests/devices. An IoMT model based on smartphones as IoT gateways is recommended (see Supplementary Figures 1,2).

Apart from REASSURED criteria, in the following, there are some challenges/points, mostly data-related issues, which should also be considered to realize the smart diagnostics of COVID-19 and other diseases with high epidemic/pandemic potential in the future:

• The Internet should be free and accessible for all in time of outbreaks/epidemics/pandemics, especially for resource-limited settings/low-income countries.
• A sufficient number of fog and cloud servers should be devoted especially for pandemics-related data to avoid network/data traffic and facilitate the process.
• Smart system architecture with unified strategies for data collection, transmission, and interpretation should be designed to integrate all above-mentioned new digital technologies (IoT, Fog, and Cloud Computing, DL, ML, NN, BA, AI, etc.) based on Industry 4.0/Healthcare 4.0 principles, into a single platform. Besides, smart diagnostics’ big data should be transcoded and purified to generate smart data for future smart services and use in the war against pandemics *via* such a platform (see Supplementary Figure 2).
• Lack of ethical guidelines, interoperability, standardization (i.e., Internet protocol version 6: IPV6), and security protocols for establishing smart diagnostics-related data is tangible; therefore, a multi-technology, multi-interface, multi-standard, and communication platform, which can integrate different communication and security standards in a unified platform *via* smartphones, is needed.

• The generation of smart pandemic data directly from home/self-users (without interruptions) is extremely needed to realize the smart diagnostics.

• Smart and alert citizens are also needed in such a war against pandemics. How to perform self-sampling, self-testing, data sharing/receiving, and safe disposal of the REASSURED-based diagnostic tests (after use) along with other pandemics-related essential educations should be trained to a large number of populations in advance *via* especial drills (similar to earthquake or fire drills).
• Multi-disciplinary and multi-cultural groups from different countries with sufficient funds in different disciplines such as medicine, engineering, (bio)chemistry, business, and information technology (IT) should be formed to tackle such problems from different angles. Such diversity and outside the box philosophy will lead to innovative and smarter solutions.

## Data Availability Statement

The original contributions presented in the study are included in the article/Supplementary Material, further inquiries can be directed to the corresponding author/s.

## Author Contributions

All authors listed have made a substantial, direct and intellectual contribution to the work, and approved it for publication.

## Conflict of Interest

The authors declare that the research was conducted in the absence of any commercial or financial relationships that could be construed as a potential conflict of interest.
