# Supplementary material for: Toward Smart Diagnostics in a Pandemic Scenario: COVID-19
Source: Front Bioeng Biotechnol. 2021 Jun 17;9:637203. doi: 10.3389/fbioe.2021.637203 (PMC8247766; doi:10.3389/fbioe.2021.637203)
Supplement: Supplementary file 1 [file Data_Sheet_1.docx]

Supplementary Material

# The proposed IoT-Fog-Cloud model for smart diagnostics

It is noteworthy that for improving the potential functions of the developed (bio)sensing test/devices, comparable versions of the proposed model has been reported via our research group (Golmohammadi et al.;Ardalan et al., 2020;Naghdi et al., 2020). The proposed model is summarized in Figure SI 1. The proposed model involves the integrations and interactions of cloud and fog, edge, and sea ( service endpoint agent) computing technologies at regional, local, and device levels, respectively(Yang, 2019). A tailored Industry 4.0 unified 5C-level architecture(Lee et al., 2015) has been adopted in our self-developed roadmap design. The proposed architecture consists of five major (either within- or between-mediating) inter-related layers with the presupposition of security to all layers using blockchain technology. For end-to-end security, a Lightweight Scalable Blockchain (LBS) security model(Dorri et al., 2019) was proposed, whereas Message-Digest Algorithm (MD5), Rivest–Shamir–Adleman (RSA) was suggested for storage purposes and Wi-Fi Protected Access (WPA), Wireless Application Protocol (WAP), Transport Layer Security (TLS) for communication purposes. Data processing and aggregation layers were designed to serve as a mediating layer for constructing smart data and additional adeptness for smart cloud services and Enterprise Resource Planning (ERP) management based on an AI. Figure SI 1 summarizes this model, whereas a detailed version of our recommended model is depicted in Figure SI 2.

As depicted in Figure SI and with more details in Figure SI 2 that follow the philosophy of knowledge pyramid (Data-Analysis-Knowledge-Wisdom) mixed with IoT-based architecture (Things-Gateway-Cloud), data from all REASSURED-based tests/devices via smartphone crowdsourcing with the help of cloud and fog servers is collected. Such data can be categorized into diagnostics, epidemiological, clinical, genetic, immunologic and symptom-relayed one in layer one as the first filtering step. Data with the help of new digital technologies such as ML, DL, BDA methods is analyzed. Through analytics procedures data is purified, filtered and transcoded for further smart solutions and applications. Such smart data generated via different types of analysis and filtering in layer two and three, will be used for not only smart services such as Internet of Medical Things (IoMT), e-Diagnostics and telemedicine but also for smart management of the pandemics via a unified ERP used by WHO or any national or international authorities. Such knowledge management system will create a pandemic wisdom and that is the philosophy behind our proposed model. Different types of decision-makers and even regular people can use this platform and even share the useful data.


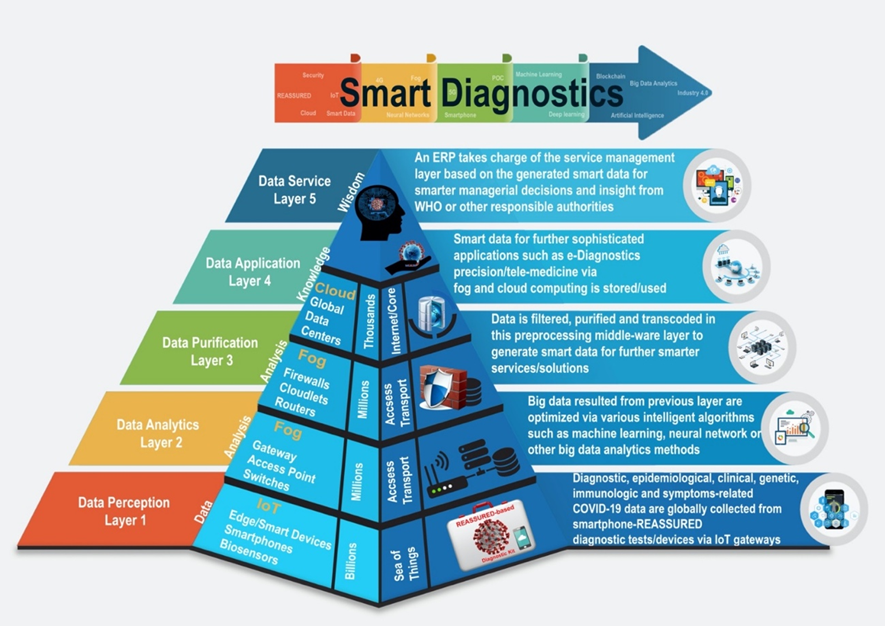


**Figure SI 1.** Proposed model of smart diagnostics. Strategies for data collection, transmission, and interpretation to integrate new digital technologies into a single platform.

#
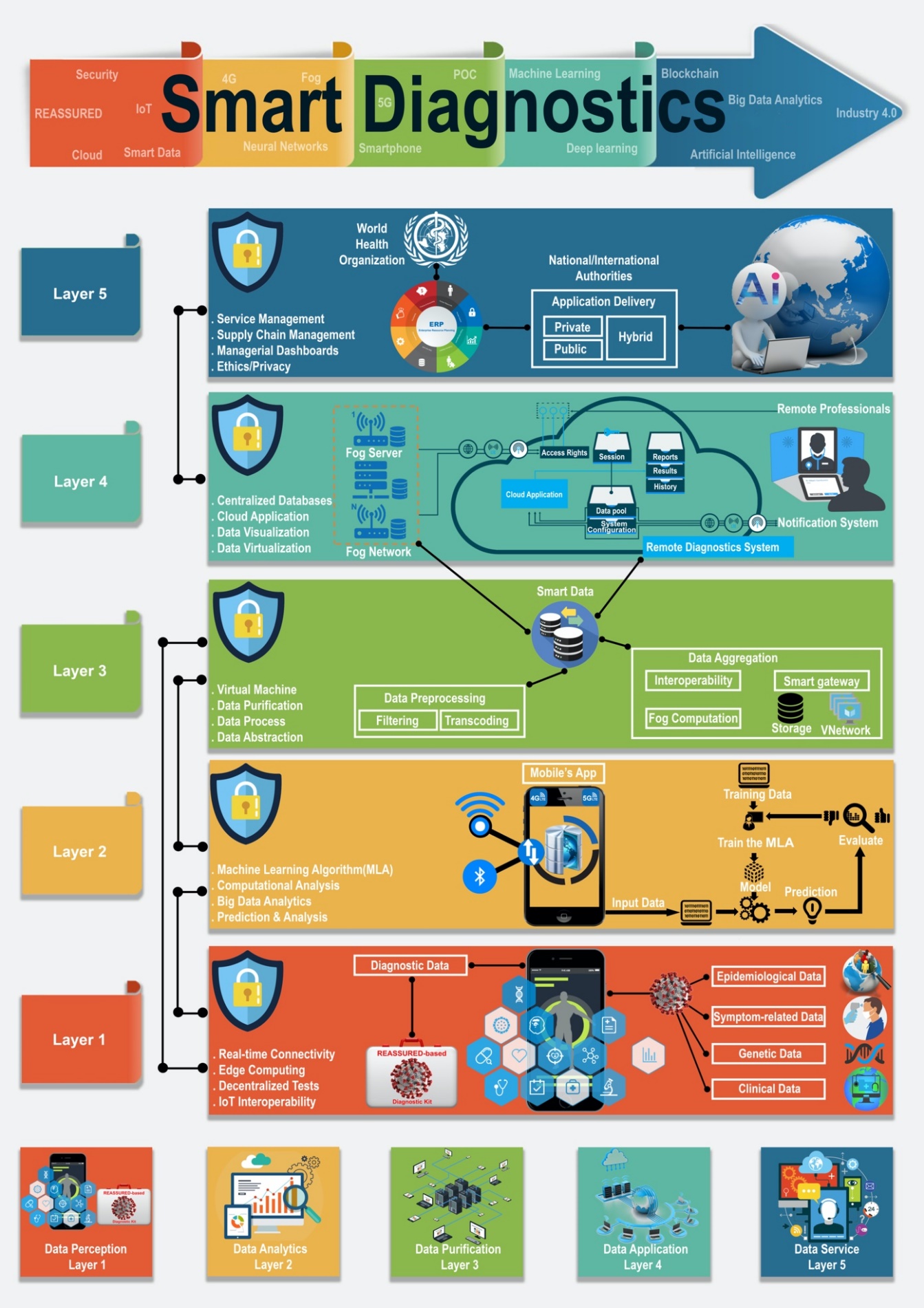


**Figure SI 2.** Proposed IoT-Fog-Cloud model for smart diagnostics: A detailed version.

**2 Acronyms List**

| 5C | Connection, Conversion, Cyber, Cognition, Configure | IoT | Internet of Things | REASSURED | Real-time connectivity, Ease of specimen collection, Affordable, Sensitive, Specific, User-friendly, Rapid and robust, Equipment-free, and Deliverable |
| --- | --- | --- | --- | --- | --- |
| AI | Artificial Intelligence | **IoAT** | Internet of Analytical Things | **RFID** | Radio-Frequency Identification |
| AR | Augmented Reality | **IoMT** | Internet of Medical Things | **RSA** | Rivest–Shamir–Adleman |
| BA | Blockchain Analysis | **IPV6** | Internet Protocol Version 6 | **RT-LAMP** | Reverse Transcription-Loop Mediated Isothermal Amplification; |
| BDA | Big data analytics | **IT** | Information Technology | **SARS** | Severe Acute Respiratory Syndrome Coronavirus |
| COVID | Corona Virus Disease | **LF** | Lateral Flow | **SARS-CoV-2** | Severe Acute Respiratory Syndrome Coronavirus 2 |
| CPS | Cyber-Physical-System | **LBS** | Lightweight Scalable Blockchain | **SEA** | Service Endpoint Agent |
| CRISPR | Clustered Regularly Interspaced Short Palindromic Repeats | **MD5** | Message-Digest Algorithm | **TLS** | Transport Layer Security |
| DL | Deep Learning | **ML** | Machine Learning | **WAP** | Wireless Application Protocol |
| ELISA | Enzyme-linked Immunosorbent Assay | **NFC** | Near Field Communication | **WHO** | World-Health-Organization |
| ERP | Enterprise Resource Planning | **NN** | Neural Network | **WPA** | Wi-Fi Protected Access |
| GDP | Gross Domestic Product | **POC** | Point-of-care | **WSN** | Wireless Network System |
| HIV | Human Immunodeficiency Virus | **QR** | Quick Response |  |  |

**3 Supporting References**

Ardalan, S., Hosseinifard, M., Vosough, M., and Golmohammadi, H. (2020). Towards smart personalized perspiration analysis: An IoT-integrated cellulose-based microfluidic wearable patch for smartphone fluorimetric multi-sensing of sweat biomarkers. *Biosensors and Bioelectronics* 168**,** 112450.

Dorri, A., Kanhere, S.S., Jurdak, R., and Gauravaram, P. (2019). LSB: A Lightweight Scalable Blockchain for IoT security and anonymity. *Journal of Parallel and Distributed Computing* 134**,** 180-197.

Golmohammadi, H., Hamzei, Z., Hosseinifard, M., and Ahmadi, S.H. Smart Fully Integrated Lab: A Smartphone‐Based Compact Miniaturized Analytical/Diagnostic Device. *Advanced Materials Technologies***,** 2000742.

Lee, J., Bagheri, B., and Kao, H.-A. (2015). A cyber-physical systems architecture for industry 4.0-based manufacturing systems. *Manufacturing letters* 3**,** 18-23.

Naghdi, T., Golmohammadi, H., Yousefi, H., Hosseinifard, M., Kostiv, U., Horák, D., and Merkoçi, A. (2020). Chitin Nanofiber Paper toward Optical (Bio) sensing Applications. *ACS Applied Materials & Interfaces* 12**,** 15538-15552.

Yang, Y. (2019). Multi-tier computing networks for intelligent IoT. *Nature Electronics* 2**,** 4-5.
